# Sarcopenia prediction using shear-wave elastography, grayscale ultrasonography, and clinical information with machine learning fusion techniques: feature-level fusion vs. score-level fusion

**DOI:** 10.1038/s41598-024-52614-2

**Published:** 2024-02-02

**Authors:** Jisook Yi, Seok Hahn, Kangrok Oh, Young Han Lee

**Affiliations:** 1 Department of Radiology, Haeundae Paik Hospital, Inje University College of Medicine, Busan, South Korea; 2 Department of Radiology, Research Institute of Radiological Science, and Center for Clinical Imaging Data Science (CCIDS), Yonsei University College of Medicine, 50-1 Yonsei-Ro, Seodaemun-Gu, Seoul, 03722 South Korea

**Keywords:** Biomarkers, Ultrasound

## Abstract

This study aimed to develop and evaluate a sarcopenia prediction model by fusing numerical features from shear-wave elastography (SWE) and gray-scale ultrasonography (GSU) examinations, using the rectus femoris muscle (RF) and categorical/numerical features related to clinical information. Both cohorts (development, 70 healthy subjects; evaluation, 81 patients) underwent ultrasonography (SWE and GSU) and computed tomography. Sarcopenia was determined using skeletal muscle index calculated from the computed tomography. Clinical and ultrasonography measurements were used to predict sarcopenia based on a linear regression model with the least absolute shrinkage and selection operator (LASSO) regularization. Furthermore, clinical and ultrasonography features were combined at the feature and score levels to improve sarcopenia prediction performance. The accuracies of LASSO were 70.57 ± 5.00–81.54 ± 4.83 (clinical) and 69.00 ± 4.52–69.73 ± 5.47 (ultrasonography). Feature-level fusion of clinical and ultrasonography (accuracy, 70.29 ± 6.63 and 83.55 ± 4.32) showed similar performance with clinical features. Score-level fusion by AdaBoost showed the best performance (accuracy, 73.43 ± 6.57–83.17 ± 5.51) in the development and evaluation cohorts, respectively. This study might suggest the potential of machine learning fusion techniques to enhance the accuracy of sarcopenia prediction models and improve clinical decision-making in patients with sarcopenia.

## Introduction

Sarcopenia is defined as a decrease in appendicular muscle mass^1^. Low muscle function was added to the 2010 revision of this definition^2^. Muscle mass is commonly measured using imaging modalities such as dual-energy X-ray absorptiometry (DXA), computed tomography (CT), magnetic resonance imaging (MRI), and ultrasonography (USG). Among these modalities, CT imaging is increasingly utilized as a standard diagnostic tool for evaluating muscle quantity and quality because muscle density loss is correlated with the degree of fat infiltration^3^.

Shear-wave elastography (SWE) USG is a novel and non-invasive functional imaging technology that offers good reproducibility and objective quantitative imaging capability. It has the potential to provide more precise and qualitative information regarding soft tissue elasticity or tissue stiffness, beyond what is possible with traditional imaging methods such as CT^4^. In a study on idiopathic inflammatory myopathy, SWE measurements of the Young’s modulus of the muscle showed substantial connections with disease activity, implying that it could be employed as a new modality for tracking disease activity^5^. Additionally, a deep convolutional neural network (DCNN) using SWE and gray-scale ultrasonography (GSU) was developed as an imaging biomarker for sarcopenia, demonstrating the potential of SWE in diagnosing and tracking muscle loss in older adults^6^.

In recent years, the prediction of sarcopenia has become a prominent research topic in musculoskeletal imaging. A recent study investigated sarcopenia prediction and assessment based on muscle mass and function using GSU and SWE in elderly patients with type 2 diabetes^4^. In another pioneering study^5^, the feasibility of SWE for sarcopenia prediction in patients with chronic obstructive pulmonary disease was explored and compared with GSU. However, segmenting regions of interest (ROI) on SWE and superimposing the reproduced images on GSU remain challenges for SWE-based sarcopenia prediction. Moreover, SWE can be difficult to quantify and ROI-based SWE measurements only provide information about a specific part of the muscle.

Utilizing ultrasound examinations incorporating GSU and SWE for sarcopenia prediction has been complemented by concurrent endeavors to explore predictive models leveraging clinical data to identify crucial risk factors. Notably, Cui et al. conducted an analysis of clinical information to evaluate sarcopenia risk specifically in 84 patients with type 2 diabetes^7^. The previous two investigations scrutinized the assessment of sarcopenia prediction by examining clinical information alongside ultrasound findings, encompassing SWE values among other parameters^4,8^.

But, there is still notable deficiency persists in the availability of comprehensive models proficiently merging both clinical data and ultrasonography features for precise predictions. One promising approach is the use of information fusion, which has been successfully applied in various classification applications^7,9^ because of its potential benefits of diversity and more accurate characterization than single-modality data^10^. To address this gap, our study proposes a novel sarcopenia prediction model that combines numerical features extracted from SWE and GSU examinations with categorical and numerical features derived from clinical information.

## Results

### Demographic characteristics

Of the development cohort, 11 subjects (15.7%) were determined as “sarcopenia” (10 men, 1 woman) and 59 (84.3%) were determined as “non-sarcopenia” (39 men, 20 women). In the evaluation cohort, 18 (22.2%) were determined as “sarcopenia” (14 men, 2 women) and 63 (77.8%) were determined as “non-sarcopenia” (16 men, 47 women). In the evaluation cohort, 20 patients (24.7%) had been diagnosed with type 2 diabetes and 5 (6.2%) had a medical history of cancer, including two cases of stomach cancer, one case of breast cancer, one case of thyroid cancer, and one case of uterine cervical cancer.

Comparisons of the clinical characteristics, GSU measurements, and SWE measurements between the two cohorts are summarized in Table 1. In the development cohort, significant differences were noted in proportion of the sex (*p* < 0.001), height (*p* < 0.001), weight (*p* = 0.024), mean thickness of RFM (*p* = 0.037), mean thickness of SCF (*p* = 0.005), and mean CSA of RF (*p* = 0.012) between the “sarcopenia” and “non-sarcopenia” groups. In the evaluation cohort, significant differences were noted in mean age (*p* = 0.004), proportion of the sex (*p* < 0.001), height (*p* = 0.004), BMI (*p* = 0.009), SMI (*p* = 0.015), mean SWV (*p* = 0.038), mean thickness of SCF (*p* = 0.005) between the “sarcopenia” and “non-sarcopenia” groups.

**Table 1:** Comparison of the characteristics of development and evaluation cohorts.

Parameters	Development cohort (n = 70)			*P*-value	Evaluation Cohort (n = 81)			*P*-value	*P*-value*
	Total (n = 70)	No sarcopenia (n = 59)	Sarcopenia (n = 11)		Total (n = 81)	No Sarcopenia (n = 63)	Sarcopenia (n = 18)		
Age (year)	45.70	45.71	45.64	0.986^a^	63.69	62.05	69.44	0.004^a^	< 0.001^a^
Gender									
Female	40	39	1	< 0.001^b^	51	47	4	< 0.001^b^	0.468^b^
Male	30	20	10		30	16	14		
Height (m)	1.66	1.64	1.74	< 0.001^a^	1.59	1.58	1.69	0.004^a^	< 0.001^a^
weight (kg)	61.13	60.17	66.28	0.024^a^	63.54	63.51	63.64	0.964^a^	0.123^a^
BMI (m^2^/kg)	22.15	22.22	21.74	0.389^a^	25.04	25.51	23.39	0.017^a^	< 0.001^a^
SMI (cm^2^/m^2^)	44.31	44.59	42.78	0.512^a^	42.41	38.16	43.62	0.015^a^	0.169^a^
Thickness of RF (cm)	2.10	2.06	2.28	0.037^a^	1.69	1.72	1.58	0.109^a^	< 0.001^a^
Thickness of SCF (cm)	0.99	1.05	0.67	0.005^a^	0.91	0.982	0.662	0.005^a^	0.254^a^
CSA of RF (cm^2^)	9.22	9.38	8.30	0.012^a^	6.41	6.55	5.93	0.209^a^	< 0.001^a^
SWV of RF (m/sec)	2.13	2.10	2.30	0.181^a^	1.97	1.91	2.18	0.077^a^	0.054^a^

*BMI, body mass index; SMI, skeletal muscle index; RF, rectus femoris muscle; SCF, subcutaneous fat layer; CSA, cross sectional area.

^a^Student T-test.

^b^chi-square test.

*Comparison between development and evaluation cohorts.

### Correlation between SMI and USG measurements

**Figure 1:**
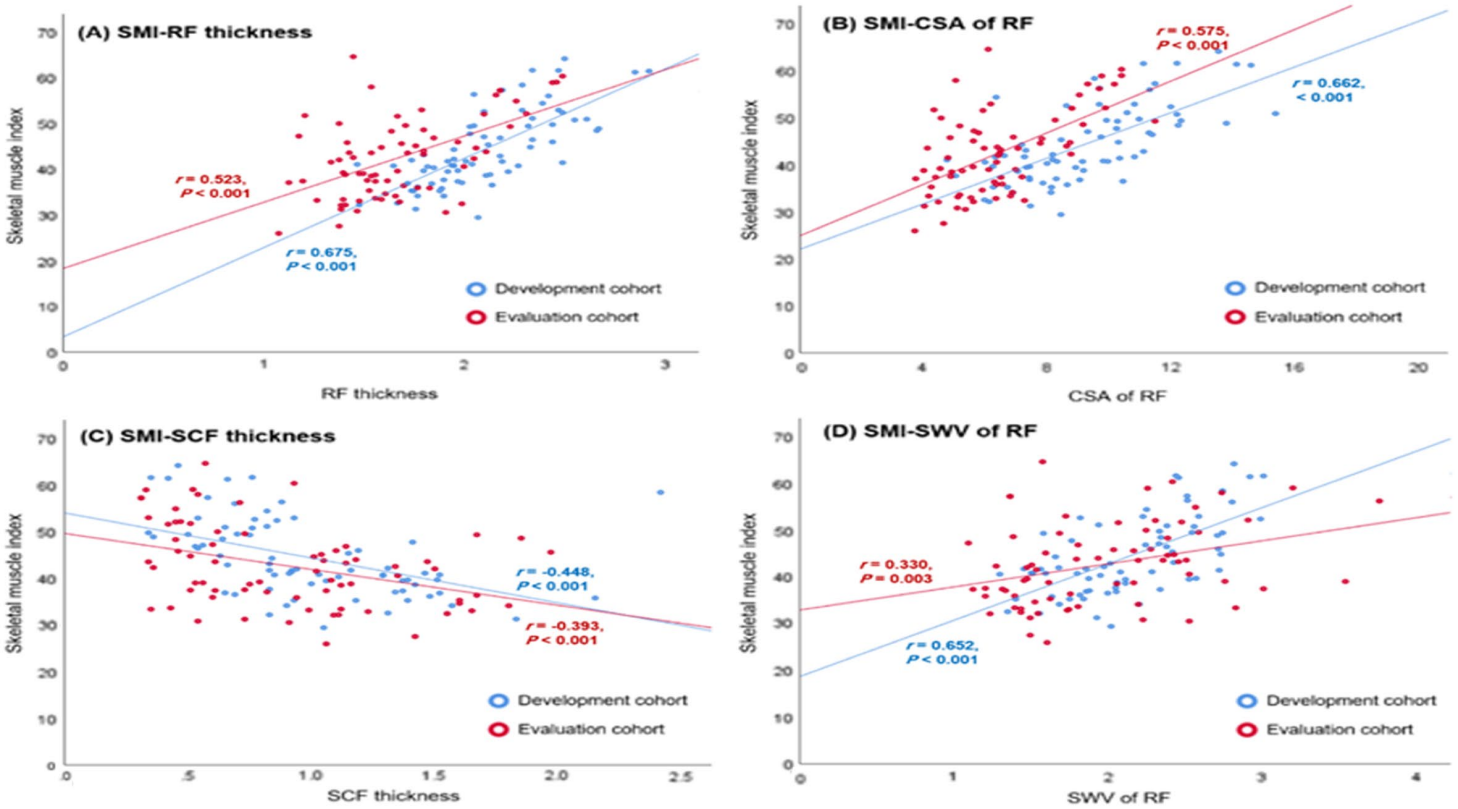
Correlation between skeletal muscle index (SMI) and ultrasonographic measurements. (**A**) Correlation between SMI and the right rectus femoris muscle (RF) thickness. (**B**) Correlation between SMI and the cross-sectional area (CSA) of RF. (**C**) Correlation between SMI and the subcutaneous fat (SCF) thickness. (**D**) Correlation between SMI and shear-wave velocity (SWV) of RF.

Figure 1 shows the correlation between the SMI and USG measurements. There was a moderate to strong positive correlation between SMI and RF thickness in both cohorts (*r* = 0.523–0.675, *p* < 0.001) (Fig. 1A). Additionally, a moderate to strong positive correlation was observed between the SMI and CSA of RF in both cohorts (*r* = 0.575–0.662, *p* < 0.001) (Fig. 1B). There was moderate negative correlation between SMI and SCF thickness of both cohorts (*r* = − 0.448 to − 0.393, *p* < 0.001) (Fig. 1C). Furthermore, the correlation between SMI and SWV of RF was strongly positive in the development cohort (*r* = 0.652, *p* < 0.001) and weakly positive in the evaluation cohort (*r* = 0.330, *p* = 0.003) (Fig. 1D).

### Sarcopenia prediction performance assessment

The sarcopenia prediction performance values obtained using the development set are summarized in Table 2. The performance values were obtained by averaging the results of leave-one-out cross-validation tests. Without combining the clinical and USG features, the clinical features showed better prediction performance (70.57% accuracy, 79.09% sensitivity, 68.98% specificity, 32.83% PPV, 94.72% NPV, and 74.53% AUC) than the USG features (69.00% accuracy, 78.18% sensitivity, 67.29% specificity, 31.26% PPV, 94.34% NPV, and 69.88% AUC). Combining the clinical and USG features at the feature level showed similar prediction performance (70.29% accuracy, 72.73% sensitivity, 69.83% specificity, 31.74% PPV, 93.40% NPV, and 68.78% AUC) with the clinical features. Among the six classifiers (sum rule, LSE, AAC optimization, RF, SVM, and AdaBoost) for the score-level fusion of clinical and USG features, the sum rule exhibited the best performance (76.71% accuracy, 73.64% sensitivity, 77.29% specificity, 38.18% PPV, 94.03% NPV, and 75.96% AUC). Score-level fusion by SR showed significantly better prediction accuracy than the clinical and USG features (*p* < 0.001 and *p* = 0.006). Score-level fusion by AdaBoost also showed better prediction accuracy than the USG features (*p* = 0.046). The rest methodologies for fusion showed no statistically significant difference from the clinical and USG features.

**Table 2:** Diagnostic performance for sarcopenia prediction in the development set (%, μ ± σ).

Models	ACC	SEN	SPE	PPV	NPV	AUC	*P*-value*	*P*-value**
Clinical	70.57 ± 5.00	79.09 ± 6.14	68.98 ± 6.83	32.83 ± 4.20	94.72 ± 0.98	74.53 ± 2.70		
USG	69.00 ± 4.52	78.18 ± 4.69	67.29 ± 5.98	31.26 ± 3.72	94.34 ± 0.87	69.88 ± 2.31		
FLF	70.29 ± 6.63	72.73 ± 9.58	69.83 ± 9.35	31.74 ± 3.78	93.40 ± 1.66	68.78 ± 4.34	0.836	0.665
SLF-SR	76.71 ± 3.81	73.64 ± 2.87	77.29 ± 4.94	38.18 ± 4.02	94.03 ± 0.44	75.96 ± 1.89	< 0.001^+^	0.006^+^
SLF-LSE	66.00 ± 6.42	85.45 ± 7.67	62.37 ± 8.55	30.35 ± 3.67	96.01 ± 1.49	74.16 ± 2.34	0.006^+^	0.224
SLF-AAC	67.29 ± 6.82	82.73 ± 10.9	64.41 ± 9.69	30.91 ± 3.85	95.51 ± 2.18	74.58 ± 2.66	0.059	0.525
SLF-RF	67.57 ± 6.39	76.36 ± 9.77	65.93 ± 8.13	30.19 ± 5.85	93.81 ± 2.13	69.57 ± 5.87	0.128	0.607
SLF-SVM	71.14 ± 13.6	46.36 ± 24.8	75.76 ± 20.1	32.23 ± 10.8	88.95 ± 2.93	48.69 ± 12.6	0.913	0.701
SLF-AB	73.43 ± 6.57	65.45 ± 16.5	74.92 ± 10.3	34.22 ± 6.04	92.41 ± 2.65	61.36 ± 6.96	0.413	0.046^+^

*μ = mean, σ = standard deviation, ACC = accuracy, SEN = sensitivity, SPE = specificity, PPV = positive predictive value, NPV = negative predictive value, and AUC = area under the receiver operating characteristics (ROC) curve, FLF = feature-level fusion, SLF-SR = score-level fusion by the sum-rule, SLF-LSE = score-level fusion by the least squares estimation, SLF-AAC = score-level fusion by area above the ROC curve optimization, SLF-RF = score-level fusion by random forest, SLF-SVM = score-level fusion by support vector machine, SLF-AB = score-level fusion by AdaBoost.

* Comparison of accuracy between the clinical features and after fusion (paired t-test).

** Comparison of accuracy between the USG features and after fusion (paired t-test).

^+^ Statistically significant.

Similar to the development set, the average sarcopenia prediction performance values measured from the evaluation set are summarized in Table 3. As in the development set, the clinical features performed better (81.54% accuracy, 67.38% sensitivity, 85.59% specificity, 61.84% PPV, 90.44% NPV, and 79.47% AUC) than the USG features (69.73% accuracy, 67.64% sensitivity, 70.32% specificity, 40.17% PPV, 88.40% NPV, and 66.83% AUC). The feature-level fusion of the clinical and USG features showed an improved prediction performance (83.55% accuracy, 60.47% sensitivity, 90.14% specificity, 67.96% PPV, 89.04% NPV, and 77.71% AUC).

**Table 3:** Diagnostic performance for sarcopenia prediction in the evaluation set (%, μ ± σ).

Models	ACC	SEN	SPE	PPV	NPV	AUC	*P*-value*	*P*-value**
Clinical	81.54 ± 4.83	67.38 ± 11.7	85.59 ± 8.96	61.84 ± 13.9	90.44 ± 2.37	79.47 ± 3.27		
USG	69.73 ± 5.47	67.64 ± 4.56	70.32 ± 7.69	40.17 ± 4.84	88.40 ± 1.59	66.83 ± 3.76		
FLF	83.55 ± 4.32	60.47 ± 10.6	90.14 ± 7.65	67.96 ± 13.0	89.04 ± 2.22	77.71 ± 3.88	< 0.001^+^	< 0.001^+^
SLF-SR	73.58 ± 4.96	68.39 ± 9.57	75.07 ± 8.42	45.94 ± 9.30	89.44 ± 2.00	74.70 ± 3.68	< 0.001^+^	< 0.001^+^
SLF-LSE	82.53 ± 6.34	60.92 ± 12.9	88.70 ± 11.3	68.83 ± 17.5	89.13 ± 2.44	78.35 ± 3.93	< 0.001^+^	< 0.001^+^
SLF-AAC	79.39 ± 7.62	61.78 ± 12.9	84.43 ± 13.0	62.39 ± 20.0	88.85 ± 2.30	76.74 ± 3.83	< 0.001^+^	< 0.001^+^
SLF-RF	77.53 ± 7.09	62.37 ± 12.4	81.86 ± 11.3	54.49 ± 15.1	88.64 ± 2.68	71.16 ± 5.63	< 0.001^+^	< 0.001^+^
SLF-SVM	80.20 ± 6.23	56.83 ± 14.1	86.87 ± 10.7	61.32 ± 15.4	87.89 ± 2.98	69.92 ± 7.84	< 0.001^+^	< 0.001^+^
SLF-AB	83.17 ± 5.51	59.97 ± 9.83	89.80 ± 8.80	67.47 ± 14.2	88.57 ± 5.14	74.78 ± 4.39	< 0.001^+^	< 0.001^+^

*μ = mean, σ = standard deviation, ACC = accuracy, SEN = sensitivity, SPE = specificity, PPV = positive predictive value, NPV = negative predictive value, and AUC = area under the receiver operating characteristics (ROC) curve, FLF = feature-level fusion, SLF-SR = score-level fusion by the sum-rule, SLF-LSE = score-level fusion by the least squares estimation, SLF-AAC = score-level fusion by area above the ROC curve optimization, SLF-RF = score-level fusion by random forest, SLF-SVM = score-level fusion by support vector machine, SLF-AB = score-level fusion by AdaBoost.

* Comparison of accuracy between the clinical features and after fusion (paired t-test).

** Comparison of accuracy between the USG features and after fusion (paired t-test).

^+^ Statistically significant.

**Figure 2:**
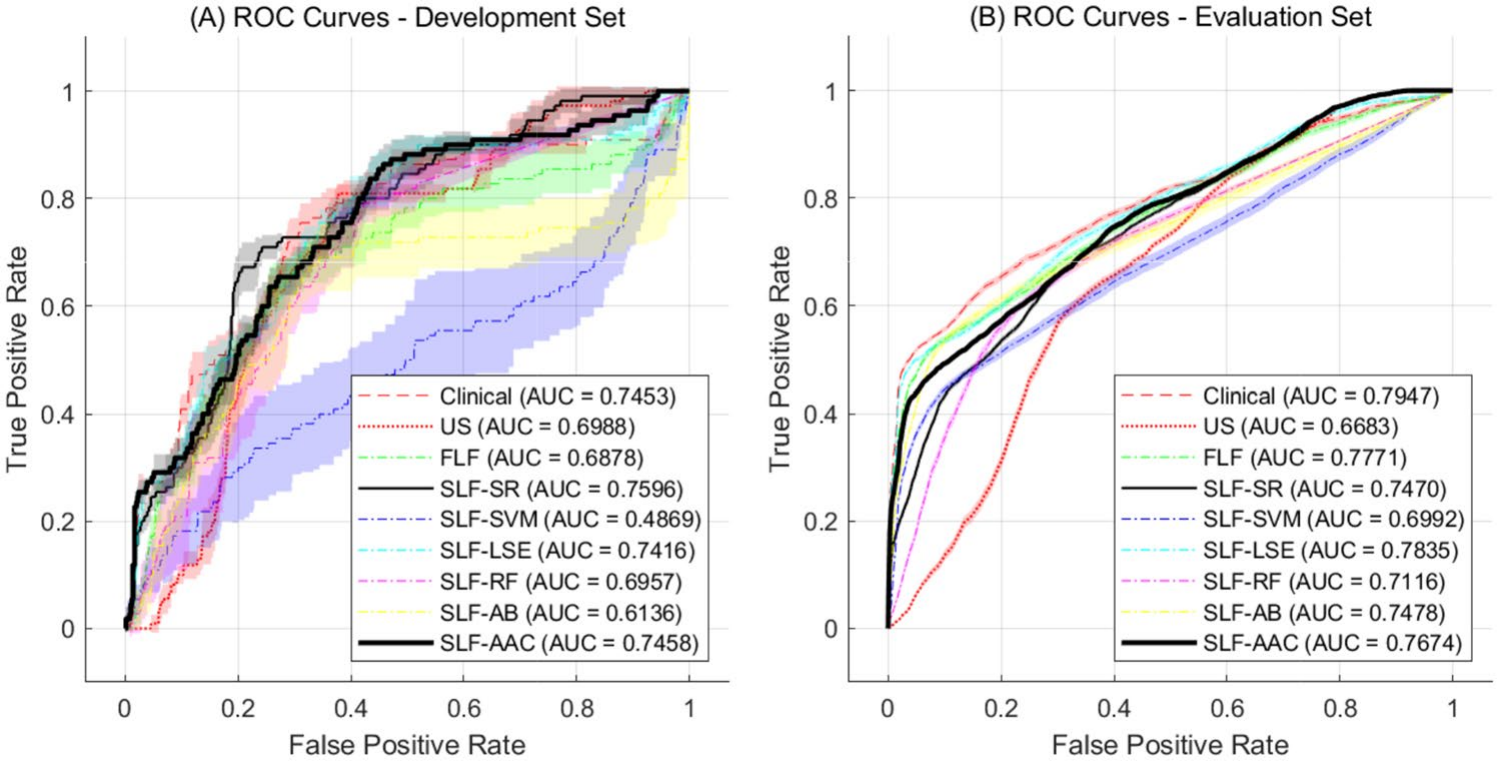
ROC curves from the experiments using the development set and evaluation set. (**A**) ROC curves from the leave-one-out cross-validation tests using the development set. (**B**) ROC curves from applying the trained models on the evaluation set.

Combining the clinical and USG features at the score level using LSE and AdaBoost showed improved prediction performance than that of the clinical and USG features (LSE: 82.53% accuracy, 60.92% sensitivity, 88.70% specificity, 68.83% PPV, 89.13 NPV, and 78.35% AUC, AdaBoost: 83.17% accuracy, 59.97% sensitivity, 89.80% specificity, 67.47% PPV, 88.57% NPV, and 74.78% AUC), where degraded prediction performance was observed for the score-level fusion using the rest methodologies. All prediction accuracy differences after fusion compared to the clinical and USG features were statistically significant (*p* < 0.001 for all cases). Figure 2 shows the test ROC curves obtained from the experiments using the development and evaluation datasets. While most methods showed similar AUC performances, except for score-level fusion based on SVM and AdaBoost, score-level fusion based on AAC minimization achieved the most reliable AUC performance in the development set (Fig. 2A). For the evaluation set, the clinical features showed the most reliable AUC performance, where the feature-level fusion and score-level fusion based on SR, LSE, and AAC showed comparable or slightly better performance than the clinical features in a partial range (Fig. 2B).

### Statistical significance tests

To validate the differences in performance before and after combining the clinical and USG features, we used the accuracy values for the paired t-test. Comparing the accuracy performance of the clinical features with the feature- and score-level fusion for the development set, the score-level fusion based on the sum rule (*p* < 0.001) outperformed the clinical features, while the score-level fusion based on LSE (*p* = 0.006) showed significantly worse performance than that based on the clinical features. In contrast, the feature-level fusion (*p* = 0.836), and score-level fusion based on AAC optimization (*p* = 0.059), RF (*p* = 0.128), SVM (*p* = 0.913), and AdaBoost (*p* = 0.413) showed no statistically significant differences. For the USG features, score-level fusion using the sum rule (*p* = 0.006) and AdaBoost (*p* = 0.046) showed a significantly better performance than the USG features, whereas the rest showed no statistically significant performance difference.

Using the evaluation set, the feature-level fusion and score-level fusion based on LSE and AdaBoost showed a significantly better accuracy performance than the clinical features (*p* < 0.001). Score-level fusion based on the sum rule, AAC optimization, RF, and SVM showed significantly worse accuracy performance than the clinical features (*p* < 0.001). Combining the clinical and USG features at the feature and score levels showed a significantly better accuracy performance (*p* < 0.001 for all methods) than that based on USG features.

## Discussion

This study investigated the usefulness of USG measurements and clinical characteristics to predict the presence of sarcopenia. Significant differences were observed in clinical characteristics (sex and height) and GSU measurement (thickness of SCF) between the “sarcopenia” and “non-sarcopenia” groups in both cohorts. Clinical features were better predictors of sarcopenia than USG features. The fusion of clinical and USG features at the feature level demonstrated accuracy performance similar to that of the clinical features in predicting sarcopenia for the development cohort and better performance for the evaluation cohort. Score-level fusion based on SR, SVM, and AdaBoost exhibited improved accuracy performance for sarcopenia prediction for the development cohort, while score-level fusion based on LSE and AdaBoost showed improved sarcopenia prediction accuracy for the evaluation cohort.

The development cohort comprises individuals with a BMI below 25 kg/m^2^ and a median age of 45.7 years. They are devoid of potential diseases that capable of influencing muscle degeneration or wastage. On the other hands, the evaluation cohort comprises a more clinically diverse group of individuals with conditions that extend beyond the typical healthy range encountered in the clinical practice, potentially impacting muscle wasting. This diversity might contribute the better accuracy in assessing sarcopenia within the evaluation cohort compared to the development cohort.

Sarcopenia is defined as the loss of muscle mass and function (strength)^11^. Muscle function depends on the contractility of muscle, which affected by muscle quality, not just by quantity (mass)^8^. USG has been used to evaluate the muscle mass and its quality without radiation exposure at a relatively low cost^12–15^. Although there is no consensus on the definition of muscle quality in assessing sarcopenia, the subjective degree of increased echo intensity (EI) on GSU has been used to evaluate muscle quality^16^. Increased echogenicity on USG reflects increased intramuscular adipose tissue, inflammation, or fibrosis and causes decreased muscle strength and altered stiffness^17^. However, Pillen et al. reported a relatively low reproducibility of subjective EI evaluation compared to the quantification of muscle EI^18^.

Recent studies have applied USG, including the SWE value of muscle, to evaluate sarcopenia and have suggested the potential of SWE values as an effective tool for clinical practice which can reflect the contractibility (function) of muscle^4,5,8^. In this study, we developed a diagnostic model for sarcopenia prediction using clinical information and USG parameters, including SWE measurements (SWV). However, unlike Chen et al., who evaluated the elderly patients with type 2 diabetes^4^, there was no statistically significant difference in the SWV values of RFM between the “sarcopenia” and “non-sarcopenia” group in either the development or evaluation cohort of the current study. This difference probably arises from the age range of the participants and the presence or absence of any comorbidities that induce muscle wasting. Nevertheless, the diagnostic performance of the current study (using the numerical value of USG) (AUC, 78.47%) was similar to a previous study (AUC, 74–84%), which utilized SWE and GSU for sarcopenia prediction using deep convolutional neural network (DCNN) learning directly from the “image itself”^13^.

Shear wave elastography (SWE), which is based on shear waves that propagate through tissues, can measure the elasticity and stiffness of tissues in the body. The quantified elasticity coefficients of SWE are represented as color-coded images or specific values from the ROI drawings that are difficult to quantify. In this study, we used both GSU and SWE for USG measurement acquisition and proposed a combination of USG measurements and clinical information at the feature and score levels. Fusing information at the feature level may increase the diversity of the acquired data. However, features obtained from different modalities may not be compatible in terms of size and discriminability. In addition, the increased feature dimensionality requires additional training data. In practice, fusing information at the score level is often preferred because of its ease of use in combining information from different modalities. Although some information loss may occur, score-level fusion is advantageous in terms of applicability. The score-level fusion approach is widely used in various classification tasks such as biometrics^19,20^ It is a suitable candidate for medical imaging applications, including ultrasound image analysis, because it provides a straightforward and practical method of combining information from different modalities. In this study, we showed that fusing clinical and USG features delivered the best prediction performance at the score level, demonstrating reliable application capability in the evaluation cohort.

This study has several limitations. First, the number of development and evaluation cohorts was relatively small for learning-based methods. However, this was a pioneering study to quantify features from multiple modalities, and it demonstrated a similar level of diagnostic performance to previous studies. Second, we defined sarcopenia based on CT images without evaluating physical performance. Further studies incorporating functional tests such as patient gait speed or handgrip strength are required to confirm these results. Finally, there was a relatively small effort to handle the data imbalance in the current study, although we adopted several classifiers. In future studies, we plan to improve the generalization capability of the sarcopenia prediction model by acquiring more datasets and adopting data augmentation and advanced learning methods such as one-shot learning to resolve small data sizes and data imbalance concerns.

In conclusion, we have successfully developed and assessed a sarcopenia prediction model. The score-level fusion approach showed a better prediction performance than the feature-level fusion approach considering both cohorts. This study highlights the potential of machine learning fusion techniques to enhance the accuracy of sarcopenia prediction models and improve clinical decision-making in patients with sarcopenia.

## Methods

### Development and evaluation data sets

**Figure 3:**
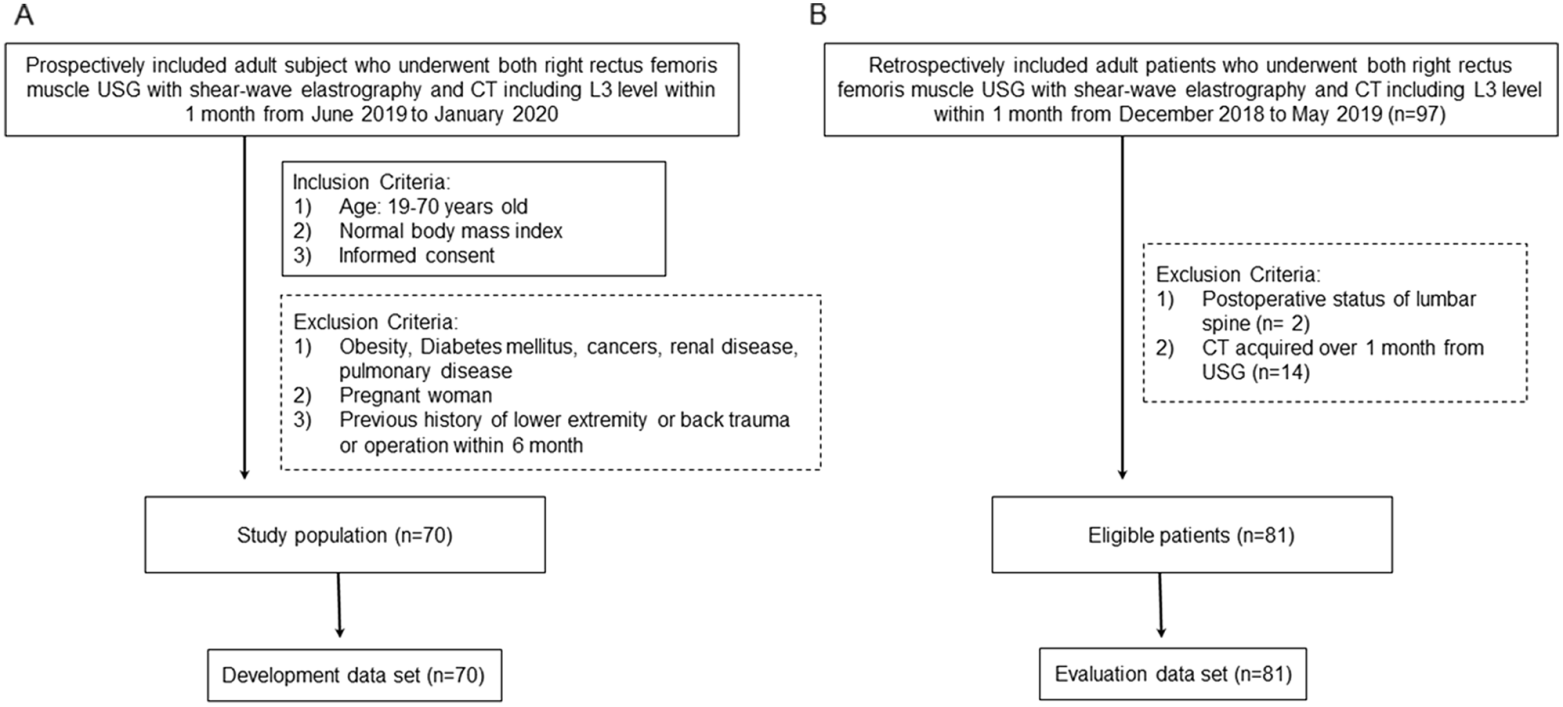
Flow diagram for development and evaluation data sets.

The development cohort was prospectively included between June 2019 and February 2020 using the following criteria: (i) 20 years ≤ age ≤ 69 years; (ii) no history of any cancer, diabetes, neuromuscular disorder, other systemic disease that might cause muscle wasting (including renal disorder and cardiopulmonary disorder); (iii) healthy body mass index (BMI [18.5–24.9 kg/m^2^]); (iv) no history of trauma of the right lower extremity; (v) no history of lumbar spine operation, and (vi) not pregnant. Seventy participants were included and none were excluded (Fig. 3A). All 70 participants underwent USG (both GSU and SWE) at the right RF and CT at the L3 level on the same day. Additionally, the evaluation cohort was retrospectively selected from those who underwent both RF USG (both GSU and SWE) and CT, including the L3 level, within 1 month between December 2018 and May 2019. The exclusion criteria for the evaluation cohort were as follows: (i) postoperative status of the lumbar spine (n = 2) and (ii) CT scans acquired over 1 month with USG (n = 14). Finally, 81 patients were included in the evaluation cohort (Fig. 3B). Clinical data including age, sex, height, weight, and BMI were collected from both cohorts. This retrospective study was approved by the institutional review board of Inje University Haeundae Paik Hospital (Approval No. 2023-05-024). The requirement for informed consent was waived. This study complied with the Declaration of Helsinki and the Health Insurance Portability and Accountability Act (HIPAA).

### Ultrasonography evaluation and analyses

**Figure 4:**
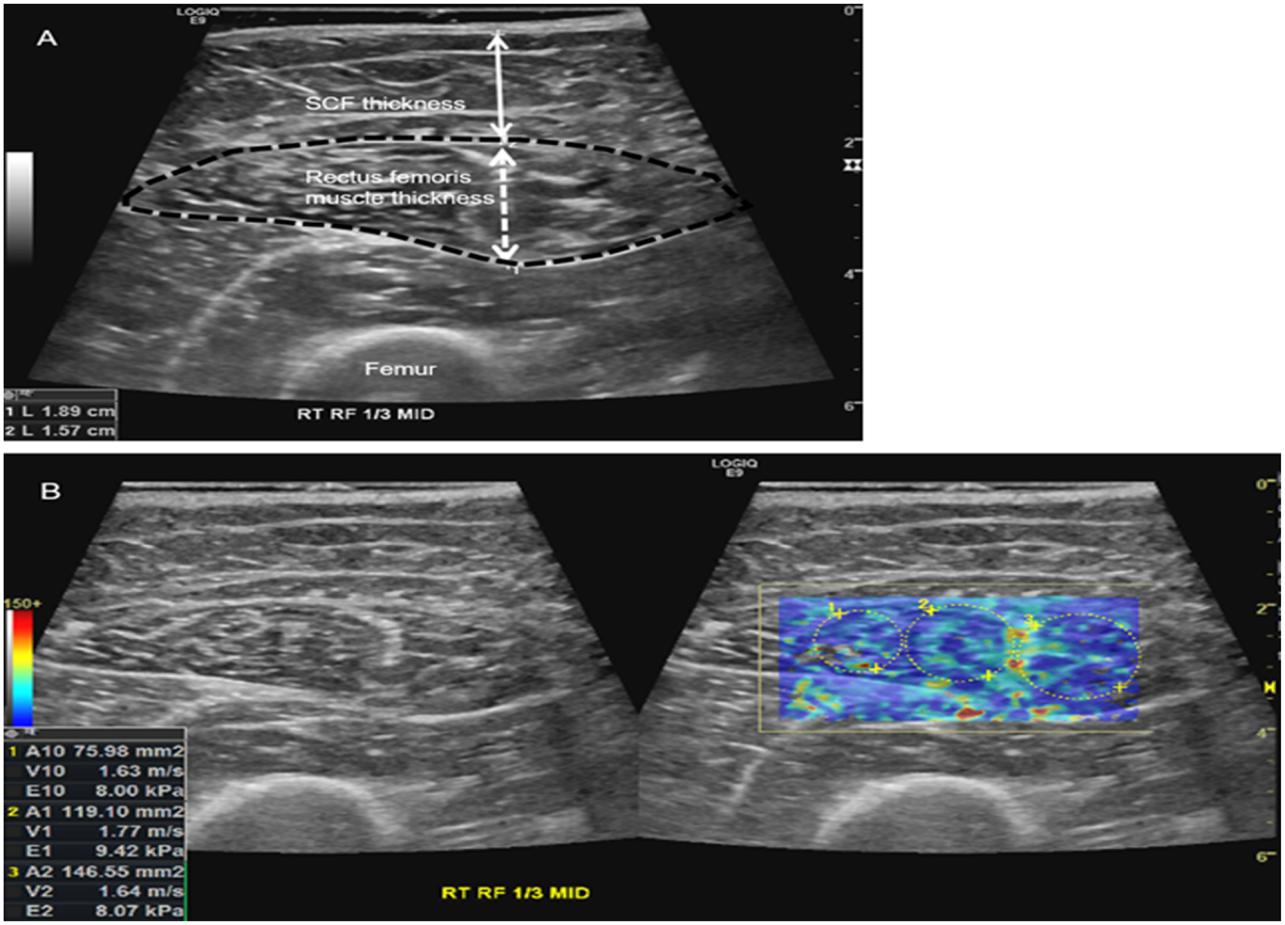
The representative image of ultrasonography measurements of mid rectus femoris muscle (RF). (**A**) On grayscale ultrasonography, the thickness of RF (white dashed double arrow), cross-sectional area of RF (black dashed line), and the thickness of subcutaneous fat layer (SCF) overlying RF (white double arrow) were measured. (**B**) On shear-wave elastography, the mean of shear wave velocity (SWV, m/sec) was measured.

All subjects underwent right RF USG (LOGIQ E9; GE Healthcare, Wauwatosa, WI, USA) using a 9 L-D linear array transducer (GE Healthcare) by a musculoskeletal fellowship-trained radiologist (with 5 years of experience, JY). The subjects were asked to lie supine with a relaxed neural ankle position. The mid-portion of the right RF was evaluated using USG, with a copious amount of gel placed on the skin to minimize external compression by the transducer. On gray-scale ultrasonography (GSU), the thicknesses of the RF and overlying subcutaneous fat (SCF), as well as the cross-sectional area (CSA) of the RF were measured (Fig. 4A). After GSU, three consecutive SWE images of RF were acquired at the same location. Three circular regions of interest (ROIs) were drawn per SWE image (Fig. 4B). The average mean shear wave velocity (SWV) (m/s) in three consecutive images was calculated for statistical analyses.

### Assessing sarcopenia on CT

CT studies were conducted at a single center using two multidetector-row CT scans on an axial plane, including the L3 vertebral level: a 64-slice system (Discovery CT 750 HD, GE Healthcare) and a 128-slice system (Definition AS + , Siemens Healthineers, Forchheim, Germany). CT images were acquired at 120 kVp and 132 mAs for both systems and reconstructed with a 5-mm slice thickness without intervals for all CT scans.

A single slice of the L3 inferior endplate level of the image was selected, and Digital Imaging and Communications in Medicine format of the image data were evaluated using the Asan J software (available at http://datasharing.aim-aicro.com/morphometry). A musculoskeletal radiologist (J.Y.), who was trained to manually trace the outline of the abdominal and back muscles (paraspinals, psoas, quadratus lumborum, transverse abdominal, abdominal internal/external oblique, and rectus abdominis), performed all CT image analyses, and the skeletal muscle area (SMA) was obtained using predetermined thresholds for the Hounsfield units (HU) on CT. The skeletal muscle index (SMI, cm^2^/m^2^) was calculated using the following formula to normalize values across patient heights:

$$ SMI = \frac{{SMA\,on\,axial\,scan\,({\text{cm}}^{2} )}}{{height\,of\,the\,patient{\text{'}}s\,({\text{m}}^{2} )}} $$

Sarcopenia was defined based on the Korean National Health and Nutrition Examination Study (KNHANES)^8^, and the cutoff value for sarcopenia was different in both sexes (sarcopenia ≤ 49 cm^2^/m^2^ for men and ≤ 31 cm^2^/m^2^ for women).

### Data preprocessing and representation

In our study, we used two types of data for sarcopenia prediction: clinical and USG features. The clinical features of sarcopenia prediction include numerical and categorical data representing the clinical characteristics of patients. These features included age, sex, height, weight, and BMI. We defined the USG features as GSU and SWE assessments, including the thickness of the mid-RF, overlying SCF, CSA of the mid-RF, and average mean SWV. All features with numerical values were rescaled using the minimum and maximum values of the training set. For binary categorical data, we assigned zero and one to the two categories. Subsequently, the clinical and USG feature vectors for sarcopenia prediction were denoted as ${x}_{C}={\left[{x}_{age},{x}_{sex},{x}_{height},{x}_{weight},{x}_{BMI}\right]}^{T}$ and ${x}_{U}={\left[{x}_{mRFT},{x}_{mRFOFT},{x}_{mRFCSA},{x}_{SWV}\right]}^{T}$, respectively. Here, ${x}_{mRFT}$, ${x}_{mRFOFT}$, ${x}_{mRFCSA}$, and ${x}_{SWV}$ are the mid-RF thickness, mid-RF overlying SCF thickness, mid-RF CSA, and average SWV, respectively.

### Sarcopenia prediction model

In this study, we proposed fusing clinical and USG information to predict sarcopenia. To verify the sarcopenia prediction performance enhancement, we compared the performance before and after combining clinical and USG features. For sarcopenia prediction using each clinical and USG feature separately, we adopted a linear regression model with least absolute shrinkage and selection operator (LASSO) regularization^10^ for each modality (clinical and USG features). For simplicity, we refer to the adopted model as the LASSO. While a ridge regression model penalizes the sum of squares of the weight coefficients to prevent overfitting^21^, LASSO estimates the weight coefficients by shrinking the sum of their absolute values to less than a fixed value. Consequently, some of the weight coefficients are forced to be zero, resulting in feature selection capability. Because of this advantage, LASSO has been applied to various regression and classification applications, including medicine^22–24^.

**Figure 5:**
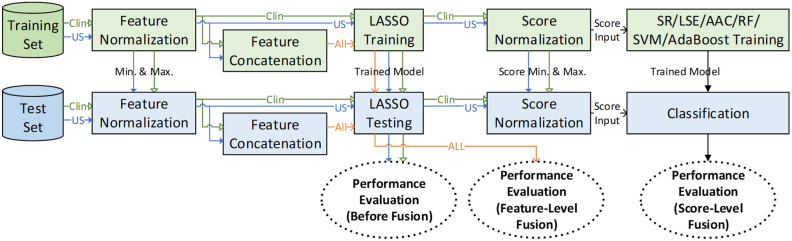
An overview of the proposed sarcopenia prediction model. LASSO is adopted for clinical and USG features and fusing them at the feature level. Additionally, score-level fusion exploits several classifiers, such as SR, LSE, AAC minimization, RF, SVM, and AdaBoost.

Information fusion approaches at various stages of pattern classification systems have been thoroughly investigated in several publications^19,25–27^. To combine the clinical and USG features, we propose fusing them at both the feature and score levels. For the feature-level fusion approach, we generated a feature vector, ${x}_{F}={\left[{x}_{C}^{T}, {x}_{U}^{T}\right]}^{T}$ which concatenated the clinical and USG feature vectors. Subsequently, we adopt LASSO for classification as in sarcopenia prediction using each feature modality. To fuse the clinical and USG features at the score level, a new vector concatenating the LASSO model outputs for each feature modality was produced, where the vector was denoted as ${x}_{S}={\left[{x}_{SC}, {x}_{SU}\right]}^{T}$. In the representation, ${x}_{SC}$ and ${x}_{SU}$ are the normalized LASSO model outputs obtained using ${x}_{C}$ and ${x}_{U}$, respectively. Then, we adopted the sum-rule^28^, least squares estimation (LSE)^29^, area above the receiver operating characteristics (ROC) curve (AAC) optimization^20^, random forest (RF) ^30^, support vector machine (SVM)^31^, and adaptive boosting (AdaBoost)^32^ as classification techniques. The score-level fusion approach can adopt various types of classifiers in the process. Therefore, we adopted several well-known classifiers for performance comparison purposes and diversity. We only provide input representations (feature vectors) because classification techniques such as LASSO, LSE, AAC optimization, RF, SVM, and AdaBoost are generic algorithms. We note that LSE and AAC search for a deterministic solution by minimizing the sum of squared errors and approximated area above a receiver operating characteristic curve, respectively, and SVM searches for a solution in an iterative manner by maximizing margin. Unlike these single classifiers, random forest and AdaBoost are ensemble classifiers based on bagging and boosting. We refer the readers to their original works ^20,29–32^ for a very detailed exploration. Figure 5 presents an overview of the proposed sarcopenia prediction model.

### Statistical analyses

The Shapiro–Wilk test was used to determine whether the data were normally distributed. Paired and independent t-tests and chi-square tests were performed to compare the clinical parameters, SMI, and USG parameters between the “sarcopenia” and “non-sarcopenia” groups and between “development” and “evaluation” cohorts. Pearson’s correlation was used to evaluate the correlation between SMI and USG measurement parameters. We also evaluated the sarcopenia prediction performance of the model in terms of accuracy, sensitivity, specificity, positive predictive value (PPV), negative predictive value (NPV), and the area under the receiver operating characteristic (ROC) curve (AUC). The optimal threshold values for the accuracy, sensitivity, specificity, PPV, and NPV were selected according to the Youden index. The threshold value is set at $max\left({r}_{SEN}+{r}_{SPE}-1\right)$, where ${r}_{SEN}$ and ${r}_{SPE}$ denote sensitivity and specificity, respectively. For each performance indicator, the average and standard deviation values were reported using the results from leave-one-out cross-validation tests for the development set. For each training set, stratified tenfold validation was performed for hyper-parameter tuning. Similarly, for the evaluation set, we reported the average and standard deviation values by applying the trained models on the evaluation set. To verify whether the performance enhancement by fusion was statistically significant, we performed a paired *t-*test^26^ using the AUC values. Statistical significance was set at *p* < 0.05. MedCalc (version 20.218; MedCalc Software, Ostend, Belgium) and MATLAB (version 9.12.0 R2022a; MathWorks Inc., Natick, Massachusetts) were used for statistical analyses.

## Data Availability

The datasets generated during and/or analyzed during the current study are available from the corresponding author on reasonable request.
